# Targeting methicillin-resistant *Staphylococcus aureus*: The comprehensive action of ent-kaurane diterpenoids on bacterial integrity

**DOI:** 10.1080/21505594.2025.2585630

**Published:** 2025-11-09

**Authors:** Yuan Yang, Xiangyun Tan, Jie Tu, Xinyu Huang, Zhenpeng Qiu, Han Xiao

**Affiliations:** a Institute of Maternal and Child Health, Wuhan Children’s Hospital (Wuhan Maternal and Child Healthcare Hospital), Tongji Medical College, Huazhong University of Science and Technology, Wuhan, People’s Republic of China; b School of Pharmacy, Hubei University of Chinese Medicine, Wuhan, People’s Republic of China; c Department of Pharmacy, the Central Hospital of Wuhan, Tongji Medical College, Huazhong University of Science and Technology, Wuhan, People’s Republic of China; d Hubei Key Laboratory of Resources and Chemistry of Chinese Medicine, Hubei University of Chinese Medicine, Wuhan, People’s Republic of China; e Hubei Shizhen Laboratory, Wuhan, People‘s Republic of China

**Keywords:** MRSA, *Siegesbeckia orientalis* L, ent-kaurane, biofilm, antibacterial

## Abstract

Methicillin-resistant *Staphylococcus aureus* (MRSA) represents a considerable challenge to global health owing to its resistance to antibiotics. In our prior research, we identified four ent-kaurane diterpenoids (compounds **1–4**) isolated from *Siegesbeckia orientalis* L., which exhibited anti-MRSA activity. Nevertheless, the precise mechanisms by which these compounds exert their effects remain to be fully elucidated. This study aims to comprehensively evaluate the anti-MRSA properties and explore the biological processes associated with the activity of compounds **1–4**. We utilized the minimum broth dilution method, electron microscopy, and membrane integrity assays to demonstrate that these compounds inhibit the growth of MRSA by disrupting cell wall and membrane structures. Additionally, crystal violet staining confirmed their efficacy in disrupting mature biofilms. In a murine model of bacteremia, the tested compounds **1–4** demonstrated a reduction in septic symptoms and exhibited favorable biosafety profiles, with compound **1** showing the most significant antibacterial effects. Transcriptomic analysis indicated that compound **1** disrupts peptidoglycan synthesis and interferes with the metabolism of cell wall precursors. Furthermore, it modulates the expression of genes associated with ion transport and membrane-related metabolic enzymes, thereby compromising the integrity of both the cell wall and the cytoplasmic membrane. In conclusion, this study systematically characterizes the anti-MRSA activity of the diterpenoid components derived from S. *orientalis* and identifies key biological processes and gene expression changes linked to their effects, and presents a promising new strategy for the development of natural anti-MRSA pharmaceuticals.

## Introduction

*Staphylococcus aureus* is a prevalent foodborne pathogen [1]. However, the overuse of antibiotics has resulted in the emergence of antibiotic-resistant strains, particularly methicillin-resistant Staphylococcus aureus (MRSA) [2]. Therefore, MRSA represents a significant threat to public health, with infections associated with high morbidity and mortality rates [3,4]. These infections can present in a variety of forms, ranging from skin and soft tissue infections to more severe conditions such as bone and joint infections, pneumonia, sepsis, and hospital-acquired infections [5–7]. The rapid proliferation and extensive antibiotic resistance of MRSA, it has become a significant clinical challenge. In response to this pressing issue, researchers have intensified their efforts over the past decade to investigate new candidate drugs in pursuit of effective treatment strategies.

The extraction of bioactive compounds from plant resources represents a significant domain of scientific inquiry [8,9], emphasizing the utilization of the pharmacological potential inherent in natural substances [10]. The genus *Siegesbeckia*, which is part of the Asteraceae family, encompasses species such as *Siegesbeckia orientalis*, *Siegesbeckia pubescens*, and *Siegesbeckia glabrescens*. These annual herbs are widely distributed across the globe and have been acknowledged for their medicinal properties, which include the capacity to alleviate wind-dampness, promote joint health, and facilitate detoxification processes in the body [11,12]. Extracts from *Siegesbeckia* are notably abundant in diterpenoid compounds, including linear diterpenes, ent-kaurane diterpenes, and abietane-type diterpenes. Ent-kaurane diterpenoids are characterized by their unique carbon skeleton, which consists of four fused rings and multiple chiral centers. These compounds demonstrate significant biological activities, including antibacterial, anti-inflammatory, and antitumor effects, thereby rendering them valuable for a range of therapeutic applications. Recent studies have identified two novel ent-kaurane diterpenoids derived from the plant *Wedelia trilobata* (L.) Hitchc, which exhibit significant antibacterial properties against *Staphylococcus aureus* [13]. Additionally, compounds possessing the ent-kaurane skeleton, sourced from Croton *tonkinensis*, have demonstrated efficacy as inhibitors of MRSA, underscoring their potential as promising antibacterial agents [14]. In our previous research [15], we extracted dried *Siegesbeckia* powder utilizing 90% ethanol, followed by successive extractions with petroleum ether, ethyl acetate, and n-butanol. The findings indicated that the ethyl acetate extract exhibited significant antibacterial activity. Consequently, we proceeded to purify and isolate compounds with a purity exceeding 98% through silica gel, C18 silica column chromatography, and high-performance liquid chromatography. The structural identification of these compounds was accomplished using mass spectrometry, nuclear magnetic resonance spectroscopy, ultraviolet-visible spectroscopy, and infrared spectroscopy. Ultimately, we identified 12 compounds, four of which were classified as ent-kaurane-type diterpenoids: compound **1** (16*β*-hydro-ent-kauran-17,19-dioic acid) [16], compound **2** (16*α*,17-dihydroxy-ent-kauran-19-oic acid) [17], compound **3** (16*β*,17,18-trihydroxy-ent-kauran-19-oic acid) [18], and compound **4** (17,18-dihydroxy-ent-kauran-19-oic acid) [19]. These compounds demonstrated sensitivity against a variety of Gram-positive bacteria, Gram-negative bacteria, and fungi, particularly exhibiting effective bactericidal activity against MRSA. However, the specific molecular mechanisms underlying their antibacterial effects require further investigation.

This study aims to evaluate compounds **1–4** in order to identify which compound exhibits the most potent antibacterial activity. By employing a combination of physiological experiments and bioinformatics tools, we seek to explore the biological processes associated with the antibacterial activity of the most effective compound. This research is expected to yield valuable insights into the application of *S. orientalis* and contribute to the development of more effective antibacterial agents to combat multidrug-resistant bacteria in the future.

## Materials and methods

### Chemicals and reagents

Peptone (Cat# LP0042) and yeast extract (Cat# LP0021) were purchased from Thermo Scientific (Waltham, USA). The crystal violet (Cat# SLBP0250V) and pentobarbital sodium salt (Cat# P3761) were purchased from Sigma Aldrich (Missouri, USA). The agar powder (Cat# BS195) was purchased from Biosharp (Hefei, China). The Bacterial Total RNA Extraction Kit (Cat# G3644), SweScript All-in-One First-Strand cDNA Synthesis SuperMix (Cat# G3333-50) and 2×Universal Blue SYBR Green qPCR Master Mix (Cat# G3326-01) were purchased from Sevier Biotechnology Co. (Wuhan, China). Vancomycin (Cat# V8050) was purchased from Solarbio Technology Ltd. (Beijing, China). SYTOX™ Green (Cat# MX4228) was purchased from Shanghai Maokang Biotechnology Co., Ltd. (Shanghai, China). Annexin V-FITC Apoptosis Detection Kit (Cat# C1062M) was purchased from Beyotime Biotechnology (Shanghai, China). 2% rabbit erythrocytes were purchased from Kewei Biotechnology Co., Ltd. (Jiangsu, China).

### Plant materials

The herb *S. orientalis* was harvested from the Anhui province of China in 2020 and was authenticated by Prof. Zhenpeng Qiu (School of Pharmacy, Hubei University of Chinese Medicine, China). A voucher specimen (No. SO-2020–03) was deposited at Institute of Maternal and Child Health, Wuhan Children’s Hospital, China. As stated in our previous study [15], the crude powder of dried *S. orientalis* was extracted using 90% ethanol. Then, extracts were obtained using petroleum ether, ethyl acetate, and n-butanol. The ethyl acetate extract was found to possess high anti-MRSA activity. Therefore, it was further isolated and purified by silica gel column, Sephadex LH-20 and ODS chromatography and preparative HPLC to obtain the compounds with a purity greater than 98%. The components obtained from the isolation and purification were structurally characterized by mass spectrometry, nuclear magnetic resonance spectroscopy, ultraviolet-visible spectra and infrared spectroscopy, finally obtained compound **1** (16*β*-hydro-ent-kauran-17,19-dioic acid), compound **2** (16*α*,17-dihydroxy-ent-kauran-19-oic acid), compound **3** (16*β*,17,18- trihydroxy-ent-kauran-19-oic acid) and compound **4** (17,18- dihydroxy-ent-kauran-19-oic acid).

### Strains and culture condition

Methicillin-resistant *Staphylococcus aureus* (strain ATCC 43,300) was obtained from the BeNa Culture Collection (Henan, China). The strain was cultured in Luria-Bertani (LB) broth (5 g·L^−1^ yeast extract, 10 g·L^−1^ sodium chloride, and 10 g·L^−1^ tryptone) at 37°C in a shaker at 200 rpm. 100 μL of resuscitated MRSA was added to 100 mL of LB medium and incubated at 200 rpm for 8–24 h. The resulting bacterial suspension was centrifuged at 4000×g for 10 min, and the precipitated bacteria were washed with 0.2 mM PBS and diluted in PBS to achieve the desired density of CFU·mL^−1^ for use in subsequent experiments. Furthermore, LB agar plate was prepared by adding 15 g agar per liter LB liquid medium. A small amount of bacterial solution was applied to LB agar plates using a trilinear method, followed by incubation at 37°C in a constant temperature incubator to observe bacterial growth.

### Minimum inhibitory concentration (MIC) and minimum bactericidal concentration (MBC) assay

The MIC was assessed utilizing the broth dilution method in a 96-well plate format. In this procedure, the compounds were serially diluted, combined with bacterial cultures, and incubated at 37°C. The MIC was defined as the lowest concentration of compounds **1–4** at which no visible bacterial growth was detected. To further ascertain the MBC, 10 μL of the culture from each well was transferred onto an agar plate and incubated at 37°C for a duration of 24 h. The MBC was defined as the lowest concentration of the compounds at which no bacterial colonies were present on the agar plate.

### Time-to-kill curve

The MRSA bacterial suspension was distributed into a 24-well plate, to which compound **1**, **2**, **3**, or **4** were added at concentrations of 1/2×MIC, 1×MIC, and 2×MIC, respectively. A bacterial suspension without any compound served as the control group. The culture plate was incubated at 37°C, and samples were collected at 0, 2, 4, 8, 16, and 24 h. Subsequently, the bacterial suspension was serially diluted using dilution factors of 10^4^, 10^5^, and 10^6^. Ten microliters of each dilution were inoculated onto agar plates and incubated at 37°C for 24 h. Following incubation, the colony count on the agar plates was determined, and the data were converted to CFU·mL^−1^. Finally, a bactericidal curve was plotted with incubation time on the x-axis and the logarithmic value of bacterial count on the y-axis to evaluate the dynamic bactericidal effects of the different compounds.

### Transmission electron microscopy (TEM) and scanning electron microscopy (SEM)

A single colony was selected from the bacterial culture plate and inoculated into 10 mL of LB culture medium, followed by incubation at 37°C with shaking at 150 rpm for 12 h. The bacterial solution was then diluted to a concentration of 1.0 × 10^5^ CFU·mL^−1^ using LB culture solution. Compound **1**, **2**, **3**, or **4** were added to MRSA standard strain bacterial fluids to achieve 1×MIC concentrations of each compound. The unadded group was used as a blank control, and 0.2% DMSO was used as a solvent control. The bacterial solutions were then incubated at 37°C, 120 rpm for 4 h with shaking. Subsequently, the bacterial cells were harvested and treated with an electron microscope fixative. TEM and SEM were performed as described previously to assess the impact of compound **1**, **2**, **3**, or **4** on the cell morphology of MRSA bacteria.

### Mature biofilm disruption assay

To evaluate the effects of compounds **1–4** on biofilms, a previously reported method was slightly modified as follows [20,21]: MRSA in the logarithmic growth phase was inoculated into sterile 96-well plates and incubated statically at 37°C for 24 h to allow biofilm formation. Subsequently, the culture supernatant was discarded, and 200 μL of fresh medium containing compounds **1**, **2**, **3**, or **4** was added to each well at final concentrations of 1/2×MIC, 1×MIC, and 2×MIC, respectively. A solvent control and a negative control were included simultaneously. After further static incubation at 37°C for 24 h, the medium was removed, and the wells were gently rinsed with sterile PBS to eliminate planktonic bacteria. Next, 4% paraformaldehyde was added to fix the biofilm cells for 20 min. After discarding the fixative, the cells were stained with 0.1% crystal violet solution at room temperature for 20 min. Following washing with PBS to remove unbound stain, 30% glacial acetic acid was added for decolorization, and the absorbance of the crystal violet solution was measured at 570 nm.

### Bacterial flow analysis

To assess the proportion of bacterial apoptosis, an Annexin V-FITC apoptosis detection kit was employed, and quantitative analysis was conducted using flow cytometry. Initially, the bacteria were cultured to a concentration of 1 × 10^6^ CFU·mL^−1^, after which compound **1**, **2**, **3**, or **4** were introduced at a concentration equivalent to 1×MIC. The cultures were incubated in a constant-temperature shaker at 37°C for a duration of 8 h. Following this incubation period, the bacterial suspension was collected and subjected to two washes with PBS buffer to eliminate any residual medium. The bacteria were subsequently resuspended in Annexin V binding buffer, to which Annexin V-FITC dye and propidium iodide were added. The resulting mixture was gently agitated and incubated in the dark at room temperature for 15 to 30 min. Finally, the samples were diluted and analyzed using the NovoCyte D2060R flow cytometer (Agilent Technologies, Santa Clara, CA, USA).

### MRSA bacterial membrane integrity assay

SYTOX™ Green is a green nucleic acid dye impermeable to membranes and selectively binds to nucleic acids with a minimum number of bases. It exhibits over 500-fold fluorescence enhancement upon binding to nucleic acids. The binding reaction, however, only takes place when the cell membrane is disrupted. Briefly, bacterial fluids treated with 1×MIC of compounds **1–4** were incubated with 3 μM SYTOX™ Green at both 4°C and 37°C for 1 h. Following incubation, 10 μL of each bacterial fluid group was aspirated and placed on glass slides. The green fluorescence was then observed with a fluorescence microscope (IX-73, Olympus, Japan) to assess the impact of compound **1**, **2**, **3**, or **4** on the cell membrane integrity of the MRSA standard strain.

### Hemolysis assay

The hemolysis assay was conducted following established protocols. Specifically, compound **1**, **2**, **3**, or **4** at varying concentrations were combined with 2% erythrocytes, respectively. PBS was the negative control, while sterile water acted as the positive control. Hemolytic activity was assessed by incubating the mixture at 37°C for 3 h and measuring the absorbance of the supernatant at 570 nm.

### In vivo anti-bacterial effects

Specific pathogen-free (SPF) female Kunming mice, aged 4–6 weeks and weighing 18–22 g, were obtained from Skibbes Bio-technology Co Ltd (Henan, China). The *in vivo* animal experiments were conducted from July to August 2024, and all animal-related procedures were conducted in strict accordance with the guidelines approved by the Animal Experimentation Committee of Hubei University of Chinese Medicine (Ethical Approval Number: HUCMS 57,038,552) and were consistent with the ARRIVE guidelines.

The *in vivo* antibacterial activity was evaluated using a mouse model of MRSA infection. Initially, and in accordance with previous studies [22], mice were injected *via* the tail vein with 0.1 mL of bacterial suspension per 20 g of body weight at concentrations ranging from 1.0 × 10^9^ to 4.0 × 10^9^ CFU·mL^−1^. Mouse survival was then monitored over a seven-day period to determine the appropriate inoculum for the infection model. The results demonstrated that a concentration of 1.0 × 10^9^ CFU·mL^−1^ represented a sublethal dose, as all mice survived throughout the observation period. In contrast, a concentration of 2.0 × 10^9^ CFU·mL^−1^ was identified as a lethal dose, resulting in significant mortality among the animals. Therefore, 2.0 × 10^9^ CFU·mL^−1^ was selected as the inoculation concentration for the subsequent experiments to establish a lethal infection model. For consistency, 0.1 mL of this bacterial suspension was administered *via* tail vein injection per 20 g of body weight.

The mice were randomly assigned to the blank control group, vancomycin (positive control) group, and drug-treated group (10 mg·kg^−1^ of compound **1**, **2**, **3**, or **4**). After 1 h of injecting the bacterial solution, the 6 groups of mice received intramuscular injections proportional to their body weight (0.1 mL per 20 g). The drugs were administered every 12 h for 3 days. The mice were closely monitored for signs of illness, and the survival rate was determined by observing the mice in each group for 7 days. At the conclusion of the experiment, mice were administered an intraperitoneal injection of a 1% pentobarbital sodium solution (at a dosage of 70 mg·kg^−1^ body weight). prepared in sterile saline. The induction of deep anesthesia was confirmed by the absence of the foot and corneal reflexes. Throughout the procedure, respiration was continuously monitored to ensure the safety of anesthesia. All procedures were conducted by professionally trained personnel in strict accordance with the animal welfare guidelines established by the American Veterinary Medical Association (AVMA). The drug dosage was determined by referring to relevant published literature [23,24] and combined with preliminary safety assessment results (no hemolysis was observed in the equivalent concentration test *in vitro*). Subsequently, major organs were harvested for paraffin embedding and stained with hematoxylin-eosin (H&E) for histological examination.

To further investigate the *in vivo* antibacterial activity of compound **1**, **2**, **3**, or **4**, we assessed the bacterial count in the blood of mice infected with MRSA. The MRSA-infected mice were randomly divided into six groups: blank control group, vancomycin (positive control) group, and drug-treated group (10 mg·kg^−1^ of compound **1**, **2**, **3**, or **4**). The drugs were administered *via* intramuscular injection. Blood samples were collected 2 h post-administration by mixing 20 μL of whole blood with 180 μL of an 8% heparin sodium solution. Subsequently, 20 μL of the resulting mixture was evenly spread onto agar plates using a bent glass rod and incubated at 37°C for 24 h. Following the incubation period, the plates were removed, and the bacterial counts in the blood of each group were quantified by counting the number of colonies.

### Sample preparation for RNA sequencing

To explore the gene expression changes in MRSA due to compound **1**, we conducted a transcriptomic analysis. Initially, MRSA was cultured to mid-log phase (OD_600_). Subsequently, the bacterial cells were treated with compound **1** at a concentration of 1/2 MIC at 37°C for 5 h. Untreated bacterial cells served as the control group. Following treatment, cells were harvested through centrifugation, washed three times with sterile PBS, and then immediately frozen in liquid nitrogen.

### RNA extraction, library construction, and sequencing

Total RNA was isolated using the Bacterial RNA Extraction Kit, with integrity and quality assessed using a Thermo NanoDrop One and an Agilent 4200 TapeStation. Ribosomal RNA was removed using the Ribo-Zero rRNA Removal Kit to purify the mRNA. Libraries were prepared following the NEBNext Ultra II Directional RNA Library Prep Kit. The quality of the constructed libraries was confirmed with fastp before sequencing on an Illumina NovaSeq^TM^ 6000 platform.

### RNA-Seq data analysis

To ensure the reliability of the analysis, the raw data underwent rigorous quality control. Following this step, all analyses were based on high-quality, clean data. The clean reads were aligned to the reference genome of the experimental species, with both genomic and gene alignments assessed for accuracy. We utilized RSEM to calculate fragments per kilobase million (FPKM) to identify significantly expressed genes [25]. For differential expression analysis, the DESeq2 and edgeR R package were employed [26,27]. Genes showing differential expression were further analyzed for KEGG pathway and GO functional enrichment, using criteria of | log2(fold change) |≥1 and FDR ≤0.05.

### Real-time fluorescence quantitative reverse transcription PCR (RT-qPCR)

Briefly, MRSA (1.0 × 10^6^ CFU·mL^−1^) was incubated for 18 h with a medium containing 1×MIC concentration of compound **1**, **2**, **3**, or **4**, respectively, under incubation conditions of 37°C and 150 rpm shaking. Bacterial precipitates were obtained by centrifugation at 4500×g for 5 min at 4°C. The RNA extraction was carried out following the instructions of the Bacterial RNA Extraction Kit. The SweScript RT II First Strand cDNA Synthesis Kit was utilized for reverse transcribing cDNA templates from RNA. The experiments were conducted using Universal Blue SYBR Green qPCR Master Mix (Service, Wuhan, China) on the CFX96 Touch Real-Time PCR Detection System (Bio-Rad), strictly following the manufacturer’s instructions [28]. Data were normalized using 16S rRNA as the reference gene. Subsequently, the relative expression level of the target gene was calculated using the 2^−ΔΔCt^ method. The primers utilized in the study can be found in Table S1.

### Statistical analysis

The data are presented as the mean ± standard deviation (SD). The results were analyzed using Prism 9 (GraphPad Software Inc., CA, USA). Treatment effects in more than two groups were assessed using one-way analysis of variance (ANOVA). Perform survival curve analysis using the log-rank (Mantel-Cox) test. All statistical analyses were conducted at a significance level of *p* < 0.05. Data acquisition and statistical analysis complied with the recommendations on experimental design and analysis in pharmacology.

## Results

### Compounds 1-4 inhibit the growth of MRSA

**Figure 1.**
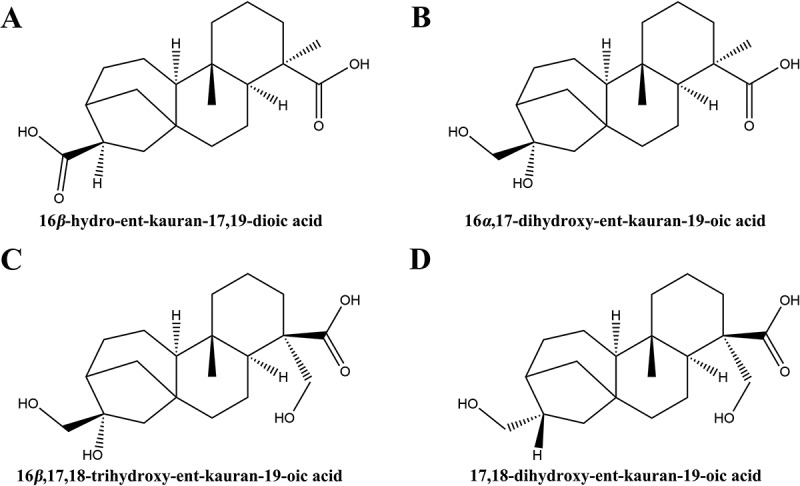
Chemical structures of compounds **1–4**. (A) Compound **1**. (B) Compound **2**. (C) compound **3**. (D) Compound **4**.

**Figure 2.**
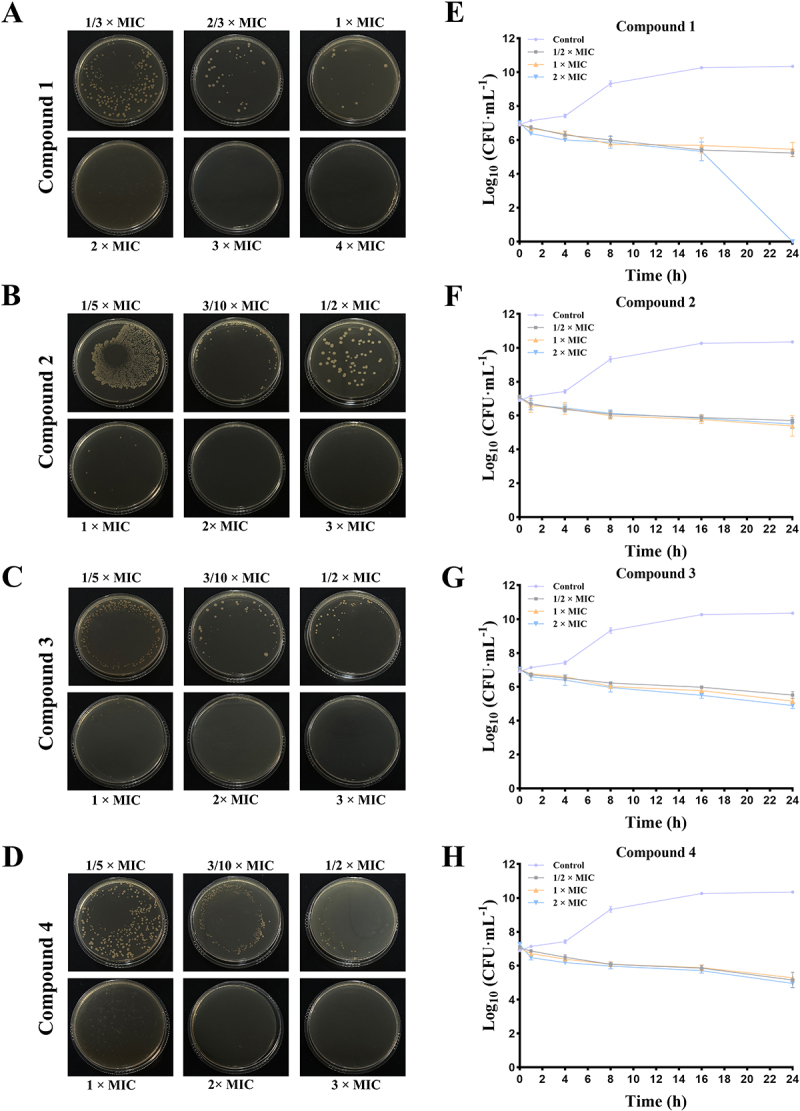
Compounds **1–4** inhibit the growth of MRSA. Determination of the minimum bactericidal concentration (MBC) for compound **1** (A), Compound **2** (B), Compound **3** (C), and compound **4** (D). Time-kill curves of compound **1** (E), Compound **2** (F), Compound **3** (G), and compound **4** (H). Three triplicate experiments were independently performed. Data were expressed as Mean ± SD.

**Figure 3.**
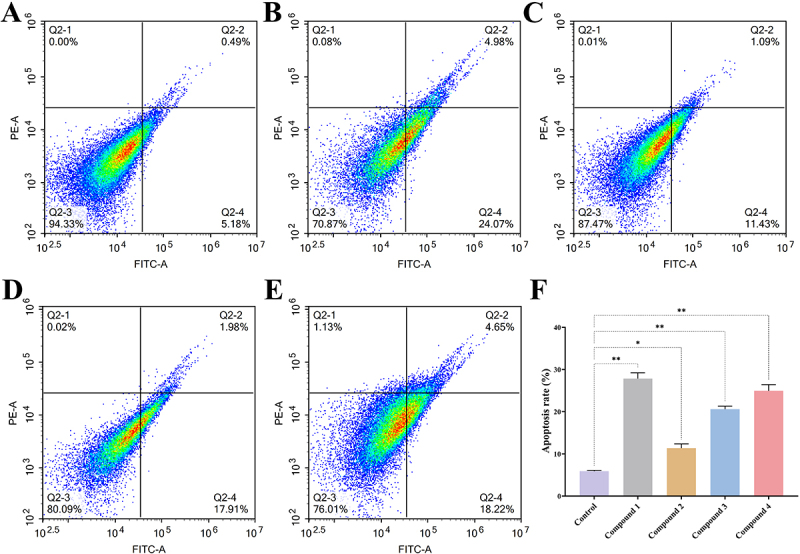
Compounds **1–4** promote MRSA death. Representative flow cytometry images showing apoptosis changes in MRSA for the control group (A) and groups treated with compound **1** (B), compound **2** (C), compound **3** (D), and compound **4** (E). (F) Quantification of cell apoptosis. Three triplicate experiments were independently performed. Data were expressed as Mean ± SD. *p < 0.05, **p < 0.01 as compared to control group.

This study builds upon our previously published research [15], specifically focusing on the antibacterial efficacy of four ent-kaurane-type diterpenoids isolated from the ethyl acetate extract of *S. orientalis* (Figure 1). In addition, the data of ^1^H NMR, ^13^C-NMR, and HREIMS of compounds **1–4** are provided in the supplementary material (Table S2). We evaluated their antibacterial efficacy against MRSA by determining the MIC and MBC using the micro-broth dilution method. The results indicated that the MIC values for compounds **1–4** were 0.12, 0.50, 0.50, and 0.25 mg·mL^−1^, respectively (Table 1), suggesting that compound **1** exhibits the most potent inhibitory effect against MRSA. Correspondingly, colony counts revealed that the MBC values were 0.24, 1, 1, and 0.5 mg·mL^−1^ (Figure 2(A–D)), further reinforcing the potent bactericidal activity of compound **1**. Additionally, time-to-kill curves of the bacteria in the presence of each compound were plotted to examine the growth and reproduction patterns of the bacteria under specific conditions. These time-to-kill curves provide insights into how each compound influences bacterial proliferation over time, thereby aiding in the elucidation of their potential mechanisms of action. In the control group, bacterial numbers exhibited slow growth between 0 and 4 h, followed by a rapid increase from 4 to 8 h, ultimately reaching the logarithmic growth phase at 16 h. In contrast, all treatment groups displayed varying degrees of bacterial growth inhibition after 2 h of exposure to the compounds, with compound **1** demonstrating the most significant inhibitory effect (Figure 2(E–H)). Notably, this inhibitory effect was positively correlated with drug concentration, suggesting its potential dose-dependent efficacy. Additionally, flow cytometry results provided further insights, revealing a significant increase in the proportion of apoptotic cells in groups treated with compound **1** and compound **4** compared to the control group (Figure 3(A–F)). This increase in apoptosis indicates that these compounds effectively inhibit bacterial growth and reproduction by inducing cell death. In conclusion, the results of our study suggest that compounds **1–4** are capable of effectively killing MRSA *in vitro*, with compound **1** exhibiting the highest efficacy. These findings underscore the potential of these compounds as promising antibacterial agents, warranting further investigation into their mechanisms of action and potential therapeutic applications.

**Table 1.** Determination of the MIC and MBC values of compounds **1–4** against MRSA.

Compounds	MIC (mg·mL^−1^)	MBC (mg·mL^−1^)
1	0.12	0.24
2	0.50	1.00
3	0.50	1.00
4	0.25	0.50

### Compounds 1-4 disrupt cell wall and membrane of MRSA

**Figure 4.**
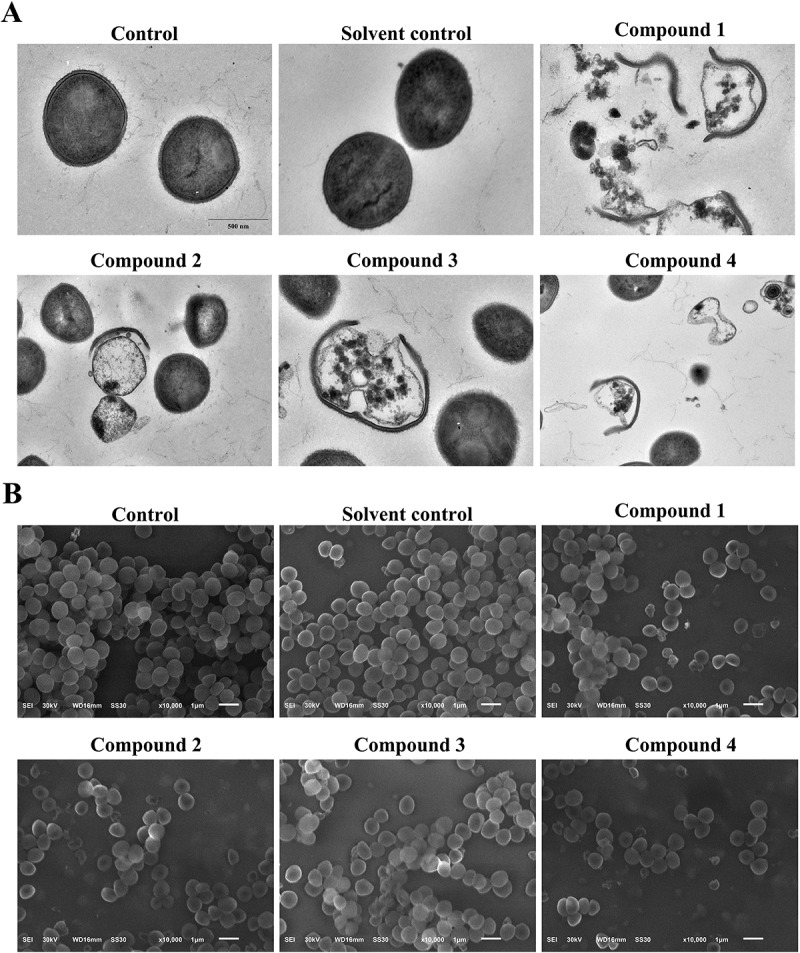
Compounds **1–4** disrupt cell wall of MRSA. Representative TEM images (A) and SEM images (B) of MRSA for the control group, solvent control group, compound **1** group, compound **2** group, compound **3** group, and compound **4** group.

**Figure 5.**
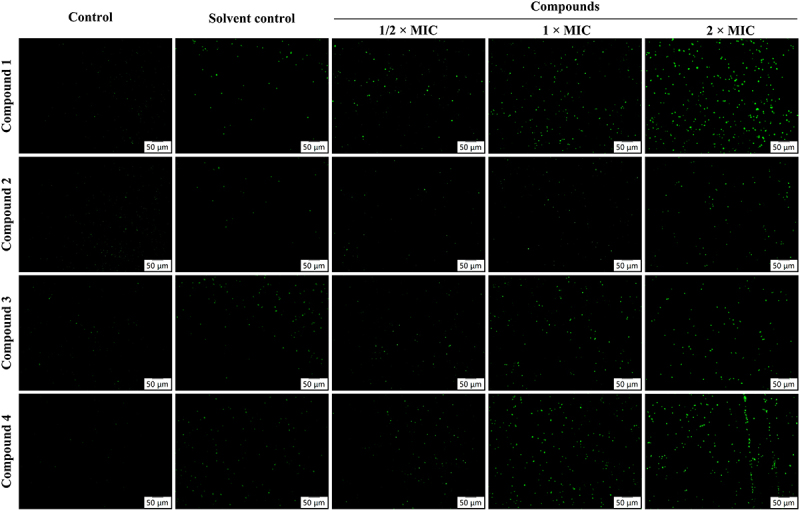
Compounds **1–4** disrupt membrane of MRSA. Microscopic images of the control group, solvent control group, compound **1** group, compound **2** group, compound **3** group, and compound **4** group stained with SYTOX™ Green, magnification: 200×, scale bar: 50 μm. Three triplicate experiments were independently performed.

The effects of compounds **1–4** on the cell wall and membrane of MRSA was assessed using advanced microscopy techniques, including SEM and TEM. In both the control and solvent control groups, the bacterial structures remained intact with well-defined cell walls and membranes. In contrast, treatment with compounds **1–4** resulted in significant alterations, including cell wall thickening, lighter coloration, indistinct membrane boundaries, and occasional bacterial rupture (Figure 4(A–B)). To further investigate the impact on MRSA cell membrane integrity, SYTOX™ Green staining was employed. This method facilitated the visualization of fluorescent spots within the cells, indicative of membrane disruption. The results demonstrated an increase in green fluorescence in the groups treated with compound **1**, **2**, **3**, or **4**, thereby illustrating their capacity to compromise MRSA cell membrane integrity in a dose-dependent manner (Figure 5). These findings suggest that compounds **1–4** possess the ability to effectively disrupt both the internal and external structures of MRSA, underscoring their potential as antibacterial agents.

### Compounds 1-4 disrupt mature biofilms of MRSA

**Figure 6.**
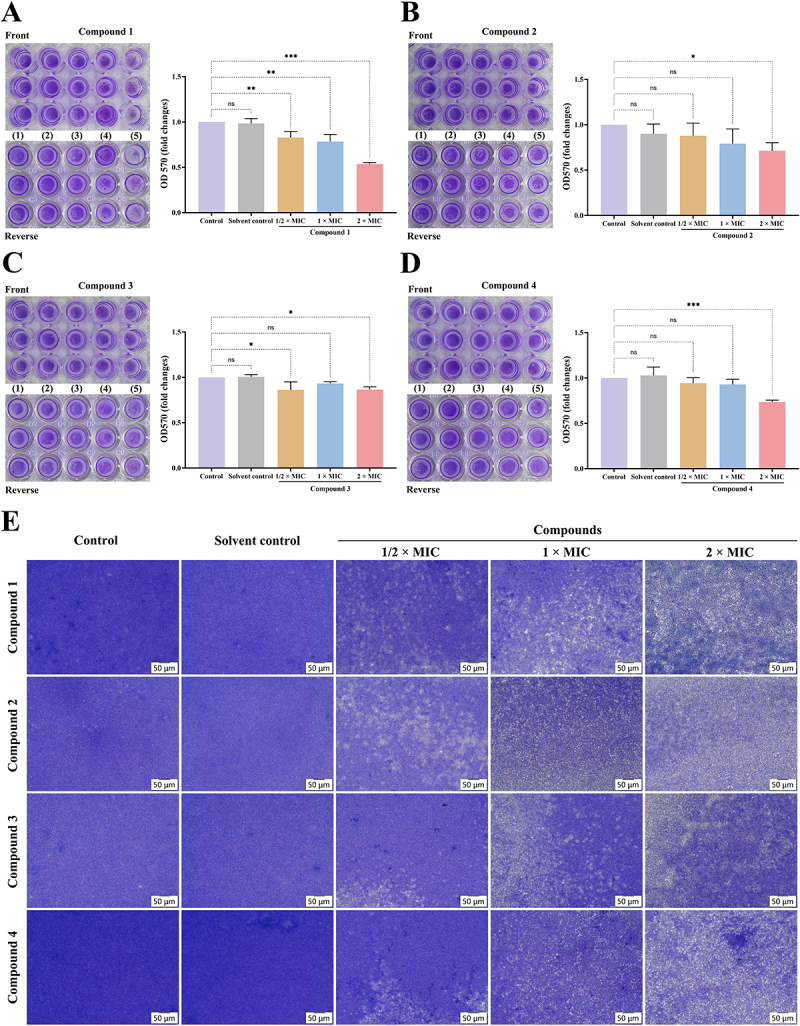
Compounds **1–4** disrupt mature biofilms of MRSA. The disruptive effects of compounds **1–4** on mature biofilms of MRSA were evaluated. The front and reverse sides of crystal violet-stained 96-well plates after treatment with each compound for 24 h, as well as the OD570 values measured after adding 30% glacial acetic acid to dissolve the stained biomass, are shown (A–D). (1) Control; (2) solvent control; (3) 1/2×MIC; (4) 1×MIC; (5) 2 ×MIC. Biofilm formation after treatment with each compound was observed under a microscope (E), magnification: 200×, scale bar: 50 μm. Three triplicate experiments were independently performed. Data were expressed as Mean ± SD. *p < 0.05, **p < 0.01, and ***p < 0.001 as compared to control group.

MRSA is recognized for its significant drug resistance and its propensity to disseminate within hospital settings. Additionally, the formation of biofilms exacerbates the challenges associated with its treatment [29]. Biofilms serve as a protective barrier for MRSA, allowing it to withstand both antibiotic therapies and immune responses, thereby heightening the risk of chronic and device-associated infections [30,31]. Consequently, identifying agents capable of disrupting mature biofilms is a crucial strategy in combating antibiotic resistance. In our investigation, visual assessments and absorbance measurements obtained from 96-well plates demonstrated a reduction in mature biofilm biomass with increasing concentrations of compound **1**, **2**, **3**, or **4** (Figure 6(A–D)). Additionally, microscopic imaging revealed that as the concentration of these compounds increased, the areas devoid of biofilm expanded, while the overall quantity of biofilm decreased (Figure 6(E)). These findings indicate that compounds **1–4** disrupt MRSA mature biofilms in a dose-dependent manner, with compound **1** exhibiting the highest efficacy.

### Compounds 1-4 with bactericidal concentration has no cytotoxicity

**Figure 7.**
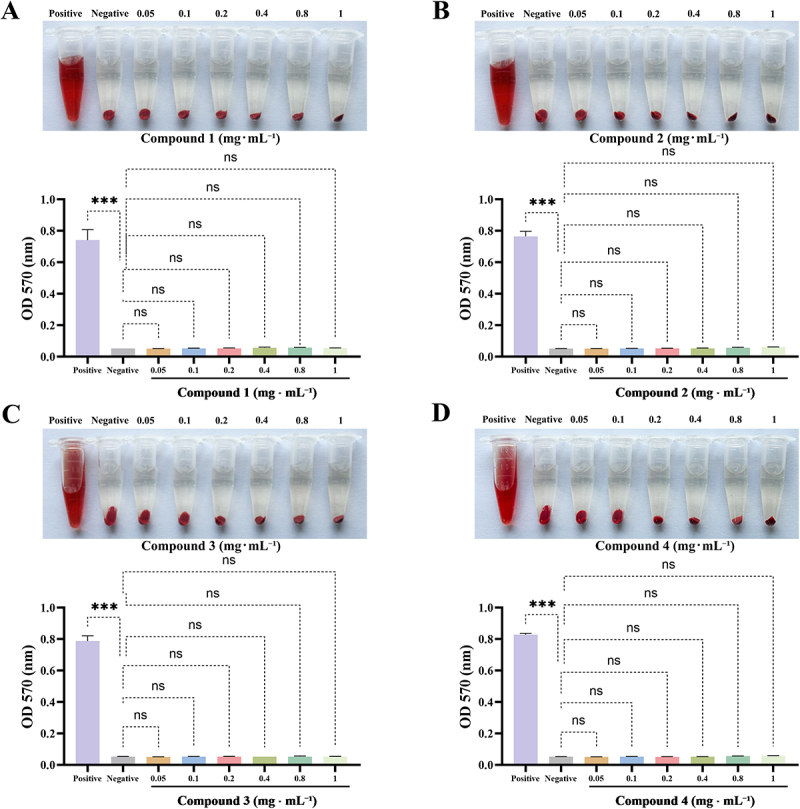
Compounds **1–4** with bactericidal concentration has no cytotoxicity. Hemolytic rates of compound **1** (A), compound **2** (B), compound **3** (C), and compound **4** (D). Three triplicate experiments were independently performed. Data were expressed as Mean ± SD. ***p < 0.001 as compared to negative group.

In addition to evaluating the efficacy of pharmaceuticals, it is essential to assess their safety profiles to determine their suitability for therapeutic applications. Hemolysis assays were conducted to examine the effects of compounds **1–4** on the integrity of red blood cell membranes. As shown in the figure, the positive control group exhibited significant hemolysis, evidenced by a color change to red in the solution, indicating membrane disruption. Statistical analysis confirmed a significant difference compared to the negative control group. In contrast, the treatment groups containing compounds **1**, **2**, **3**, or **4** showed no signs of hemolysis, even at elevated concentrations (Figure 7(A–D)). Statistical analysis revealed no significant differences compared to the negative control group. These results suggest that these compounds have a negligible effect on the stability of red blood cell membranes. This observation indicates a favorable biosafety profile, suggesting that these compounds may be safe for application without inflicting harm to red blood cells.

### Compounds 1-4 protect mice from MRSA infection

**Figure 8.**
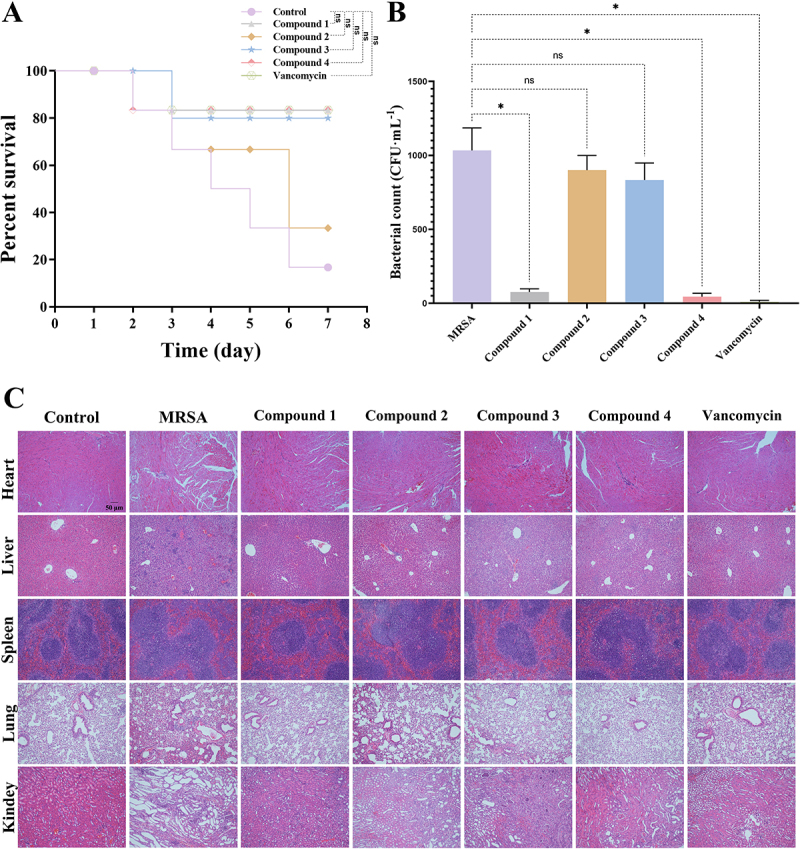
Compounds **1–4** protect mice from MRSA infection. (A) Survival rates of bacteremia mice in each group (*n* = 7). (B) Bacterial loads in the blood of bacteremia mice in each group (*n* = 6). (C) Representative H&E staining images of the heart, liver, spleen, lungs, and kidneys of bacteremia mice in each group, magnification: 200×, scale bar: 50 μm. Data were expressed as Mean ± SD. *p < 0.05 as compared to MRSA group.

A singular *in vitro* activity test is insufficient for a comprehensive evaluation of an antibacterial properties of a drug. Therefore, this study employed a mouse bacteremia model to further assess the *in vivo* antibacterial efficacy of compounds **1**, **2**, **3**, and **4**. In this model, the survival rate of mice in the control group was 28.5%. Treatment with vancomycin and compounds **1**, **3**, and **4** increased the survival rate to 85.7%, while treatment with compound **2** resulted in a survival rate of 57.1% (Figure 8(A)). In comparison to the model group, mice treated with compound **1**, compound **4**, or vancomycin demonstrated significant recovery, as evidenced by improvements in clinical symptoms, including reduced lethargy, hunched posture, and piloerection. Furthermore, the bacterial load in the bloodstream was markedly diminished in these treatment groups, underscoring their therapeutic efficacy (Figure 8(B)). A histopathological examination utilizing H&E staining was conducted on major organs, including the heart, liver, spleen, lungs, and kidneys, to evaluate pathological changes. Notable inflammatory infiltration was observed in the organs of the model group, reflecting the consequences of MRSA infection. For example, H&E staining of the heart revealed disorganized myocardial fibers, infiltration of inflammatory cells, damage to myocardial cells, and vascular congestion. The liver exhibited a disorganized arrangement of hepatocytes, pronounced inflammatory cell infiltration, and congested hepatic sinusoids. The spleen displayed varied nuclear morphology with some indications of damage, while lung tissue demonstrated substantial inflammatory cell infiltration, thickened alveolar septa, and narrowed or absent alveolar spaces. The kidneys exhibited tubular damage, interstitial edema, inflammatory cell infiltration, as well as cellular degeneration and necrosis (Figure 8(C)). Importantly, organ damage in the groups treated with compound **1** or vancomycin showed a trend toward reduction. In conclusion, these findings suggest that compound **1** possesses robust antibacterial activity *in vivo* in mice, providing a promising foundation for further exploration of its potential therapeutic applications.

### Transcriptome analysis of antimicrobial mechanisms of compound 1

**Figure 9.**
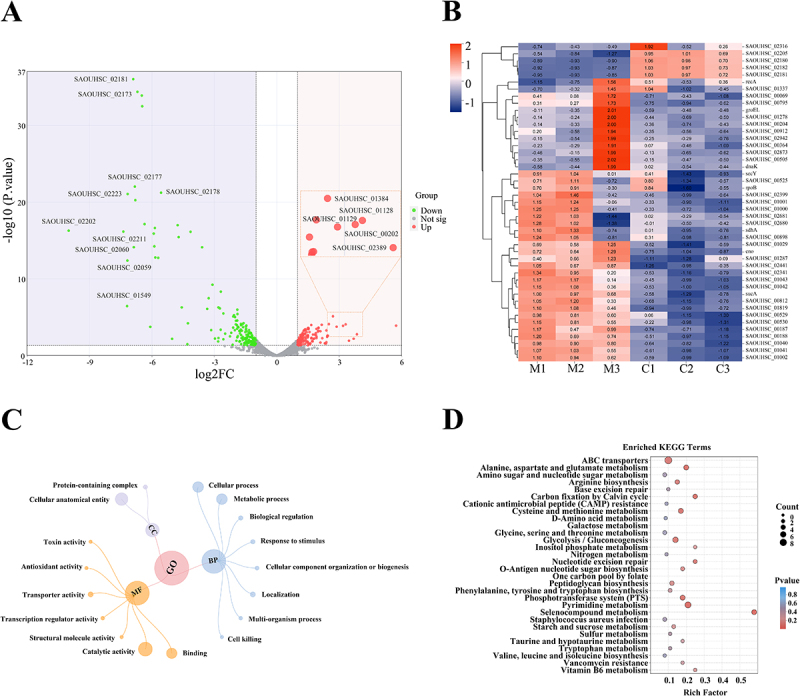
Transcriptome analysis of the effect of compound **1** on MRSA gene expression. Analysis of differentially expressed genes (DEGs) between the compound **1** group and the control group. (A) Volcano map. (B) Up- and downregulated genes according to GO functional annotation. M1-3: MRSA group, C1-3: compound **1** group. (C) GO enrichment of total DEGs. (D) KEGG analysis of the pathways in which the DEGs were enriched.

Compound **1** has exhibited significant antibacterial activity against MRSA in both *in vitro* and *in vivo* studies. To investigate the molecular changes underlying its antibacterial activity, we performed transcriptomic analysis to assess genome-wide transcriptional alterations in MRSA treated with compound **1**. The significantly affected biochemical pathways identified through these gene expression changes provided insights into the compound’s potential mechanism of action. The library construction and sequencing yielded comprehensive results, demonstrating a balanced nucleotide distribution across all samples (Table S3). The analysis identified 282 genes with significant differential expression between the compound **1**-treated and control groups. Among these, 154 genes were found to be upregulated, while 128 genes were downregulated (Figure 9(A,B)). The ten most significantly upregulated genes (Table 2) are primarily associated with MRSA’s stress defense mechanisms. Notable examples include SAOUHSC_02211 (phi PVL orf 50-like protein), SAOUHSC_01549 (transcriptional activator rinB-like protein), and SAOUHSC_02173 (amidase). Additionally, several genes critical for physiological functions were downregulated, including SAOUHSC_02389 (cation efflux family protein), SAOUHSC_01128 (ornithine carbamoyltransferase), and SAOUHSC_01129 (carbamate kinase). These findings suggest that compound **1** may exert its antibacterial effects by disrupting MRSA’s stress response mechanisms and impairing essential physiological functions, thereby inhibiting the survival and proliferation of the bacteria.

**Table 2.** The top ten up- and down- regulated DEGs.

Categary	Gene_ID	log_2_FC	FDR	Gene_Name	Gene_description
UP	SAOUHSC_02202	−9.997640351	9.30E-15	SAOUHSC_02202	hypothetical protein
	SAOUHSC_02211	−7.367064551	1.13E-14	SAOUHSC_02211	phi PVL orf 50-like protein
	SAOUHSC_01549	−7.189123862	2.92E-05	SAOUHSC_01549	transcriptional activator rinB-like protein
	SAOUHSC_02059	−7.173539933	4.01E-11	SAOUHSC_02059	phi PVL orf 52-like protein
	SAOUHSC_02178	−7.161909144	2.68E-19	SAOUHSC_02178	phi PVL orf 22-like protein
	SAOUHSC_02181	−6.893337554	2.03E-33	SAOUHSC_02181	phi PVL orfs 18–19-like protein
	SAOUHSC_02060	−6.86571752	8.67E-13	SAOUHSC_02060	phi PVL orf 51-like protein
	SAOUHSC_02177	−6.818110053	4.00E-20	SAOUHSC_02177	hypothetical protein
	SAOUHSC_02223	−6.792662298	1.49E-18	SAOUHSC_02223	phi PVL orf 39-like protein
	SAOUHSC_02173	−6.690907423	4.56E-32	SAOUHSC_02173	amidase
Down	SAOUHSC_02389	3.853151648	0.033703009	SAOUHSC_02389	cation efflux family protein
	SAOUHSC_01128	3.313901115	0.003922609	SAOUHSC_01128	ornithine carbamoyltransferase
	SAOUHSC_00202	3.187662243	0.000134416	SAOUHSC_00202	hypothetical protein
	SAOUHSC_01129	2.877519475	0.00657459	SAOUHSC_01129	carbamate kinase
	SAOUHSC_01384	2.702320814	0.000461462	SAOUHSC_01384	PhoU family transcriptional regulator
	SAOUHSC_01130	2.505124512	0.003920229	SAOUHSC_01130	hypothetical protein
	SAOUHSC_01278	2.458119335	0.045615711	SAOUHSC_01278	aerobic glycerol-3-phosphate dehydrogenase
	SAOUHSC_01452	2.432532107	0.046356778	SAOUHSC_01452	alanine dehydrogenase
	SAOUHSC_01671	2.385760908	0.014110601	SAOUHSC_01671	diacylglycerol kinase
	SAOUHSC_01389	2.164708017	0.004406718	SAOUHSC_01389	phosphate ABC transporter substrate-binding protein

The GO enrichment analysis provides a comprehensive understanding of the biological significance of the DEGs identified following treatment with compound **1**. The analysis revealed significantly enriched GO terms (*p*-adj < 0.05) for both upregulated and downregulated DEGs, emphasizing key categories across three ontological domains: biological processes (BP), cellular components (CC), and molecular functions (MF) (Figure 9(C)). In the BP ontology, the most significant categories identified were metabolic process (GO:0008152) and cellular process (GO:0009987). These findings imply that compound **1** may influence fundamental biological activities within the cell, potentially disrupting normal metabolic and cellular functions in MRSA. For the CC ontology, notable enrichment was observed in the categories of protein-containing complex (GO:0032991) and cellular anatomical entity (GO:0110165). This suggests that compound **1** impacts critical structural and organizational features of the cell, which could be essential for the survival and functionality of MRSA. Within the framework of the MF ontology, the DEGs were predominantly linked to catalytic activity (GO:0003824) and binding (GO:0005488). These functions are vital for cellular biochemical interactions and reactions, suggesting that compound **1** may disrupt essential enzymatic activities and molecular interactions in MRSA. Overall, the GO enrichment analysis offers a more profound understanding of the intricate effects of compound **1** on MRSA, highlighting its potential to interfere with critical biological processes and cellular functions.

KEGG pathway enrichment analysis was utilized to categorize DEGs into specific biological pathways. The analysis revealed the top 30 pathways significantly enriched among the DEGs, with Selenocompound metabolism (sao00450) and Pyrimidine metabolism (sao00240) emerging as the most prominent. These pathways play a critical role in the metabolic processes and indicate substantial disruption caused by compound **1**. Additional noteworthy pathways included Alanine, aspartate, and glutamate metabolism (sao00250), Carbon fixation *via* the Calvin cycle (sao00710), the Phosphotransferase system (PTS) (sao02060), Cysteine and methionine metabolism (sao00270), and Glycolysis/Gluconeogenesis (sao00010) (Figure 9(D)). The involvement of these pathways suggests that compound **1** exerts a multifaceted influence on the metabolic and cellular functions of MRSA, potentially contributing to its antibacterial properties.

**Figure 10.**
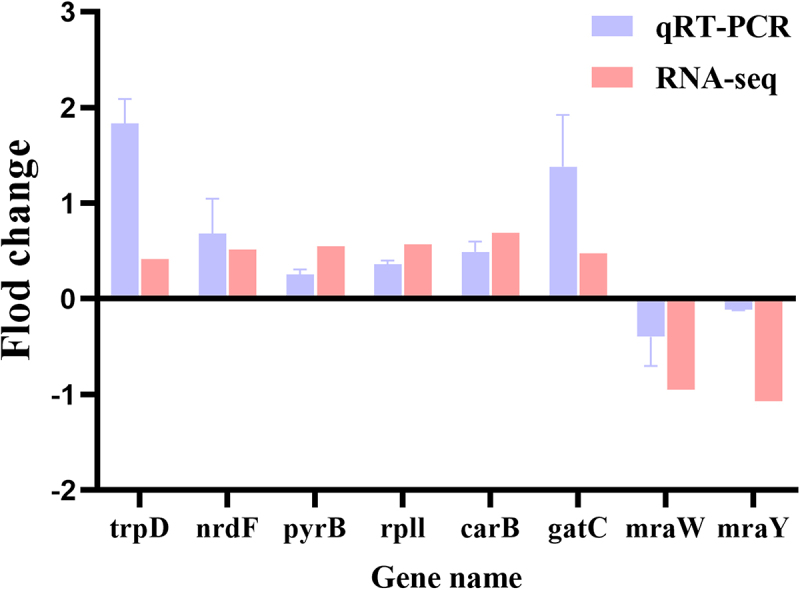
Validation of RNA sequencing data by RT-PCR. Data were expressed as Mean ± SD.

To validate the RNA-Seq data, qRT-PCR analysis was performed on eight randomly selected DEGs. The expression levels obtained from qRT-PCR were found to be consistent with the RNA-Seq results (Figure 10). This consistency serves to confirm the reliability and accuracy of the RNA-Seq analysis, thereby reinforcing the integrity of the study’s findings. Overall, the alignment of qRT-PCR and RNA-Seq data supports the potential of compound **1** as an effective antibacterial agent, primarily through its influence on key metabolic and cellular pathways in MRSA. This comprehensive approach enhances our understanding of the transcriptional regulatory changes underlying the antibacterial activity of compound **1** and highlights its potential application in combating antibiotic-resistant bacteria.

## Discussion

MRSA is a drug-resistant pathogen that is prevalent in various natural environments and is a significant contributor to invasive infections [3]. It poses considerable risks in both healthcare and community contexts, leading to conditions such as pneumonia, sepsis, and surgical site infections [32]. The resistance of MRSA to commonly utilized antibiotics, in conjunction with its high virulence, presents a formidable challenge to global public health. This situation underscores the urgent necessity for the development of novel antibacterial agents to mitigate resistance, enhance patient outcomes, and preserve the long-term efficacy of anti-infective therapies. Ent-kaurane diterpenoids demonstrate a variety of biological activities, particularly their antibacterial properties, which have attracted significant attention in recent years. Prior research has predominantly focused on bacterial species that exhibit sensitivity to these compounds, including Mycobacterium tuberculosis and methicillin-resistant MRSA [13,33,34]. Nevertheless, the precise mechanisms by which these compounds exert their antibacterial effects are not yet fully elucidated and require further investigation. In this study, we present the inaugural report on the antibacterial activity of kaurane-type diterpenoid compounds **1–4**, which were isolated from the ethyl acetate extract of *S. orientalis*. The compounds exhibited substantial inhibition of bacterial growth, effectively alleviated symptoms in septic mice *in vivo*, and demonstrated favorable biosafety profiles. Notably, compound **1** displayed the most potent antibacterial activity, surpassing the efficacy of the other three compounds in both *in vitro* and *in vivo* assessments. To investigate the gene expression changes associated with the antibacterial activity of compound **1**, a transcriptome analysis was conducted. The findings indicated that compound **1** primarily inhibits the synthesis of cell wall precursors, impact transporter proteins, and modify energy metabolism. This disruption ultimately leads to the suppression of MRSA. This study underscores the potential of ent-kaurane-type diterpenoids as promising candidates in the battle against MRSA, presenting a hopeful avenue for addressing this pressing global health challenge.

As fundamental structures supporting bacterial activity and pathogenicity, the cell wall and cytoplasmic membrane are essential for maintaining cell morphology, osmotic balance, and intracellular homeostasis [35–37]. Our study utilized SEM and TEM imaging to demonstrate that treatment with compounds **1–4** led to bacterial cytoplasmic vacuolization, detachment of the cytoplasm from the cell wall, cell lysis, and the release of intracellular contents. These observations indicate substantial damage to the structural integrity of the cell wall and membrane. Additionally, SYTOX™ Green staining was utilized to evaluate the effects of compounds **1–4** on the MRSA cell membrane. The results revealed a significant increase in fluorescence signals in the treated group, thereby confirming the compromise of membrane integrity. It is important to note that the precise molecular mechanisms underlying these structural disruptions have yet to be elucidated. Specifically, it remains unclear whether compounds **1–4** selectively interact with key bacterial components, such as peptidoglycan (PGN) in the cell wall or membrane phospholipids – including phosphatidylethanolamine (PE), phosphatidylglycerol (PG), and cardiolipin (CL). Such interactions could potentially compromise the structural integrity of the bacterial cell envelope, thereby contributing to the observed antibacterial effects. Given these uncertainties, further investigation is warranted. Future experiments could employ microdilution checkerboard assays, in which the compounds are co-incubated with varying concentrations of PGN and relevant phospholipids. Observing changes in the MIC would provide indirect evidence of potential interactions. Furthermore, isothermal titration calorimetry (ITC) could be utilized to directly measure and quantify the binding affinity and thermodynamic properties between compounds **1–4** and bacterial cell wall or membrane components [38]. Taken together, these complementary approaches would yield more direct evidence regarding the interaction patterns and help clarify the molecular basis by which these compounds exert their antibacterial activity.

Notably, the disruption of the cytoplasmic membrane and cell wall in MRSA results in instability within its biofilm structure, thereby diminishing its capacity for formation and reducing drug resistance. This disruption impedes bacterial adhesion and signal transduction, resulting in decreased biofilm stability. Consequently, antimicrobials can penetrate more effectively, which lowers the biofilm’s invasiveness and resistance, thereby creating favorable conditions for the resolution of infections. Crystal violet staining revealed that compounds **1–4** disrupted mature biofilms in a dose-dependent manner. In conclusion, these findings suggest that at elevated concentrations, compounds **1–4**, especially compound **1**, significantly compromised the permeability and integrity of the cytoplasmic membrane while concurrently disrupted mature biofilms.

Compounds **1–4** demonstrated promising antibacterial activity *in vitro*. However, reliance on a single *in vitro* assay is insufficient for a comprehensive evaluation of a drug’s efficacy. Consequently, a mouse bacteremia model was established to assess the *in vivo* antibacterial activity of these compounds. Prior to conducting *in vivo* experiments, the hemolytic activity of compounds **1–4** was evaluated, revealing satisfactory biosafety for all compounds. The *in vivo* results indicated a significant increase in the survival rate of septic mice treated with vancomycin, compound **1**, compound **3**, or compound **4**, accompanied by notable improvements in clinical symptoms. Furthermore, the bacterial load in the blood was significantly reduced in the groups treated with compound **1**, compound **4**, or vancomycin. Histological examination through H&E staining revealed severe inflammatory infiltration in the major organs of septic mice; however, significant improvement in organ damage was observed in the treatment groups receiving compound **1** and vancomycin. In conclusion, these findings suggest that compound **1** exhibits excellent antibacterial activity *in vivo* within a mouse model.

The variations in functional groups and stereochemistry among compounds **1–4** may significantly influence their biological activity, electronic distribution, and binding specificity and affinity to particular bacterial targets, resulting in differing antibacterial efficacies. For instance, compound **1** is characterized by the presence of two carboxyl groups. These carboxyl groups exhibit high polarity, which can enhance the compound’s solubility in aqueous environments, thereby facilitating its transport within biological systems and its interaction with bacterial targets [39]. Furthermore, an increased number of carboxyl groups may promote interactions between compound **1** and positively charged entities on bacterial cell membranes, potentially disrupting membrane functions such as permeability. This disruption could lead to the leakage of intracellular substances and adversely affect bacterial metabolism, thereby augmenting antibacterial activity [39]. Additionally, the acidic nature of carboxyl groups enables them to release hydrogen ions (H^+^) under physiological conditions, resulting in a negative charge that enhances binding to specific targets on the bacterial surface through electrostatic interactions, further amplifying the antibacterial effect [40]. This suggests the potential for optimizing such compounds through chemical synthesis or biotechnological approaches in the future, aimed at improving their antibacterial activity and bioavailability.

This study builds upon the remarkable antibacterial properties of compound **1** by utilizing transcriptome technology to investigate the molecular-level alterations induced by the compound’s effects on bacterial cells. The findings indicate that in the group treated with compound **1**, there is a notable upregulation of genes associated with the synthesis of cell wall precursors, including the PTS system transporter subunit IIA (SAOUHSC_01430). Furthermore, there is an increase in the expression of genes involved in the regulation of cell wall hydrolases, such as the murein hydrolase regulator LrgA (SAOUHSC_00232) [41,42]. Additionally, KEGG pathway analysis revealed that compound **1** significantly influenced several pathways, including Peptidoglycan Biosynthesis [43,44], Amino Sugar and Nucleotide Sugar Metabolism, ABC Transporters [45], and PTS. The data indicate that compound **1** predominantly disrupts the synthesis of bacterial cell wall precursors, which leads to a compromised and destabilized cell wall structure. This disruption ultimately culminates in the rupture and death of bacterial cells.

The analysis of DEGs indicated that the expression of the gene encoding the ion transport-related protein, monovalent cation/H^+^ antiporter subunit F (SAOUHSC_00884), was significantly downregulated. This downregulation may adversely affect the cell’s capacity to reverse-transport monovalent cations and hydrogen ions, potentially resulting in their accumulation within the cellular environment. Furthermore, the expression of the cation efflux family protein (SAOUHSC_02389) was also diminished, which may further impede cation expulsion and disrupt the ion concentration gradient, thereby compromising intracellular homeostasis. Significant alterations were noted in genes related to membrane-associated enzymes. For example, the downregulation of diacylglycerol kinase (SAOUHSC_01671) resulted in a reduction of phosphatidic acid synthesis, which adversely impacted membrane fluidity and stability [46]. In contrast, the upregulation of peptidoglycan hydrolase (SAOUHSC_02170) facilitated cell wall remodeling, thereby increasing structural stress on the cytoplasmic membrane. Furthermore, the expression of genes associated with transport proteins, including ABC transporters (SAOUHSC_00175, SAOUHSC_00176) and the PTS system transporter subunit IIBC (SAOUHSC_02661), was found to be upregulated. This upregulation enhances the transport of carbohydrates and solutes, thereby addressing the demands of energy metabolism [47,48]. However, it may also pose challenges to the membrane’s capacity for substance exchange. Additionally, the downregulation of the PhoU family transcriptional regulator (SAOUHSC_01384) may further compromise the structural stability and signal transduction processes of the cytoplasmic membrane. The cumulative effect of these systemic alterations in gene expression undermined the integrity and functionality of the cytoplasmic membrane, ultimately resulting in physiological disruption and mortality in the bacterial cells.

Furthermore, bioinformatics analyses indicate that the upregulation of the murein hydrolase regulator LrgA (SAOUHSC_00232) may inhibit cell wall hydrolysis, thereby reducing bacterial adhesion and aggregation. Furthermore, the upregulation of the trans-sulfuration enzyme family protein (SAOUHSC_00340) and bifunctional homocysteine S-methyltransferase (SAOUHSC_00339) could disrupt sulfur metabolism and methylation pathways, thereby affecting intracellular metabolic balance. Additionally, the increased expression of the multiple sugar-binding transport ATP-binding protein (SAOUHSC_00175) and extracellular solute-binding protein (SAOUHSC_00176) may interfere with sugar transport and the extracellular environment, hindering the synthesis of extracellular polymers. KEGG pathway analysis further elucidated that the drug disrupts bacterial biofilm formation through various pathways, including the PTS system, ABC transporters, peptidoglycan synthesis, amino sugar and nucleotide sugar metabolism, as well as starch and sucrose metabolism. These systems and pathways are essential for biofilm formation. The PTS and starch/sucrose metabolism provide the energy and carbon sources necessary for cellular metabolic activities and the synthesis of extracellular polymers. ABC transporters regulate material exchange, maintaining appropriate ion concentrations and nutrient supply [49,50]. Peptidoglycan synthesis influences cell wall structure and stability, promoting cell adhesion and aggregation [51]. Finally, amino sugar and nucleotide sugar metabolism supports the synthesis of extracellular polysaccharides, thereby enhancing bacterial adhesion and resistance. In summary, the findings indicate that compound **1** interferes with essential biochemical pathways, thereby undermining both the structural integrity and functionality of the biofilm. This disruption ultimately hinders bacterial colonization and dissemination.

In summary, we conducted a comprehensive assessment of the antibacterial activity of abietane diterpenoid compounds **1–4**, which were screened, isolated, and identified from the ethyl acetate extract of *S. orientalis*. Furthermore, transcriptome analysis was employed to identify transcriptional perturbations associated with the antibacterial activity of compound **1**, with a focus on the affected biological processes and pathways. The results indicated that compound **1** disrupts the structural integrity of bacterial cell walls and membranes, as well as the stability of mature biofilms, by targeting critical pathways, including cell wall precursor synthesis, transport proteins, membrane-associated enzymes, the PTS, ABC transporters, and peptidoglycan synthesis. However, the precise molecular targets of compound **1** within these pathways, as well as its pharmacokinetic profile *in vivo*, have yet to be fully elucidated. Therefore, future research should prioritize validating its specific molecular targets, clarifying its mechanisms of action in living organisms, and systematically assessing its potential applications. These efforts will be crucial for advancing compound 1 from laboratory research to clinical or practical use.

## Supplementary Material

Supplementary information.docx

Author Checklist.pdf

## Data Availability

The data that support the findings of this study are openly available in figshare at https://doi.org/10.6084/m9.figshare.29095703.v3, reference number [52]. The raw sequence data reported in this paper have been deposited in the Genome Sequence Archive [53] in National Genomics Data Center [54], China National Center for Bioinformation/Beijing Institute of Genomics, Chinese Academy of Sciences (GSA: CRA026576) that are publicly accessible at https://ngdc.cncb.ac.cn/gsa, reference number [55].
