# Supplementary material for: Targeting methicillin-resistant Staphylococcus aureus: The comprehensive action of ent-kaurane diterpenoids on bacterial integrity
Source: Virulence. 2025 Nov 9;16(1):2585630. doi: 10.1080/21505594.2025.2585630 (PMC12622317; doi:10.1080/21505594.2025.2585630)
Supplement: Supplementary information.docx [file KVIR_A_2585630_SM9205.docx]

**Supplementary materials**

**Targeting Methicillin-resistant *Staphylococcus aureus*: The Comprehensive Action of Ent-Kaurane Diterpenoids on Bacterial Integrity**

**Table S1** Sequences of primers used in quantitative real-time PCR (qPCR)

| **Gene** | **Sequences (5′ to 3′)** | **Length (bp)** | |
| --- | --- | --- | --- |
| 16sRNA | Forward: GAGAGAAGGTGGGGATGACGT | | 217 |
|  | Reverse: AGGCCCGGGAACGTATTCAC | |  |
| trpD | Forward: GCGGCACAGGTGGTGACAAG | | 80 |
|  | Reverse: TTTTACGCCAGCACTTGCTACAAC | |  |
| nrdF | Forward: TGGCGATGATGGAGCAAATACAC | | 293 |
|  | Reverse: ACCTGATGTCGTCATTTTCCCTTG | |  |
| pyrB | Forward: AGGCATGGGCTTGCAGAAGAAAC | | 130 |
|  | Reverse: CTCTATTCACAGGTGCCGGATGC | |  |
| rpll | Forward: CGTTGAGTTAACTTCAGCTGGTTCA | | 114 |
|  | Reverse: ACTTTAGGAGCTCCGTCTACTAATTCT | |  |
| carB | Forward: TTAACGTGCCACAGCCACAAGG | | 125 |
|  | Reverse: TTCCATTGCGCGACCACCTAATAC | |  |
| gatC | Forward: CGTGAAGAAGTTGAGCATATCGC | | 144 |
|  | Reverse: TGTAGGTTCAACGCCTTCTGTATC | |  |
| mraW | Forward: AGCTCGAAGAATCGAAGCACATCG | | 109 |
|  | Reverse: TCCGCCTTTTCTTCTTGCTTTTGC | |  |
| mraY | Forward: AGATGGATTAGCAACTGGACTGTC | | 78 |
|  | Reverse: GCCGTTTCTCCTAACACAAAGC | |  |

The Data of ^1^H NMR and ^13^C-NMR of compounds 1-4 from *Siegesbeckia orientalis* L. were shown as follows:

**16*β*-hydro-ent-kauran-17,19-dioic acid (1)**. White amorphous powder. ^1^H NMR (400MHz, CD_3_OD) *δ*_H_: 2.62 (1H, dd, *J* = 8.6, 6.3 Hz, C_16_-CH), 2.42 (brs, m, C_13_-CH), 2.12 (1H, d, C_3_-CH_2_*_α_*), 1.91 (1H, m, C_1_-CH_2_*_α_*), 1.84 (1H, m, C_2_-CH_2_*_α_*), 1.58 (1H, m, C_15_-CH_2_*_α_*), 1.42 (1H, m, C_2_-CH_2_*_β_*), 1.18 (3H, s, C_18_­-CH_3_), 1.09 (1H, m, C_15_-CH_2_*_β_*), 1.04 (1H, m, C_3_-CH_2_*_β_*), 1.00 (1H, m, C_14_­-CH), 0.95 (3H, s, C_20_­-CH_3_), 0.85 (1H, ddd, *J* = 13.6, 13.2, 4.0Hz, C_1_-CH_2_*_β_*). The data of ^13^C-NMR was showed in Table S2. HREIMS *m/z* 357.2040 [M + Na]^+^ (calcd for C_20_H_30_O_4_Na, 357.2041).

**16*α*,17-dihydroxy-ent-kauran-19-oic acid (2)**. White amorphous powder. ^1^H NMR (400MHz, DMSO-*d_6_*) *δ*_H_: 11.94 (1H, brs, C_19_-COOH), 3.50 (1H, d, *J* = 11.1Hz, C­_17_-CH_2_*_α_*), 3.39 (1H, d, *J* = 10.5Hz, C_17_-CH_2_*_β_*), 2.00 (1H, d, C_3_-CH_2_*_α_*), 1.88 (1H, brs, C_13_-CH), 1.78 (1H, m, C_1_-CH_2_*_α_*), 1.09 (3H, s, C_18_-CH_3_), 0.99 (1H, m, C_3_-CH_2_*_β_*), 0.87 (3H, s, C_20_-CH_3_), 0.75 (1H, m, C_1_-CH_2_*_β_*). The data of ^13^C-NMR was showed in Table S2. HREIMS *m/z* 359.2185 [M + Na]^+^ (calcd for C_20_H_32_O_4_Na, 359.2186).

**16*β*,17,18-trihydroxy-ent-kauran-19-oic acid (3)**. White amorphous powder. ^1^H NMR (400MHz, DMSO-*d_6_*) δ_H_: 11.89 (1H, brs, C_19_-COOH), 3.50 (1H, d, *J* = 11.1Hz, C­_17_-CH_2α_), 3.43 (1H, m, C_18_-CH), 3.39 (1H, d, *J* = 10.5Hz, C_17_-CH_2_*_β_*), 1.97 (1H, d, C_3_-CH_2_*_α_*), 1.87 (1H, brs, C_13_-CH), 1.78 (1H, m, C_1_-CH_2_*_α_*), 1.03 (1H, m, C_3_-CH_2_*_β_*), 0.87 (3H, s, C_20_-CH_3_), 0.77 (1H, m, C_1_-CH_2_*_β_*). The data of ^13^C-NMR was showed in Table S2. HREIMS *m/z* 375.2133 [M + Na]^+^ (calcd for C_20_H_32_O_5_Na, 375.2134).

**17,18-dihydroxy-ent-kauran-19-oic acid (4)**. White amorphous powder. ^1^H NMR (400MHz, CD_3_OD) *δ*_H_: 3.59 (1H, d, *J* = 11.3Hz, C­_18_-CH_2α_), 3.44 (1H, d, *J* = 10.5Hz, C_18_-CH_2_*_β_*), 3.29 (2H, m, C_17_-CH_2_), 2.07 (1H, m, C_3_-CH_2_*_α_*), 2.01 (1H, brs, C_13_-CH), 1.78 (1H, m, C_1_-CH_2_*_α_*), 1.71 (1H, m, C_15_-CH_2_*_α_*), 1.11 (1H, dd, *J* = 13.5, 4.3Hz, C_3_-CH_2_*_β_*), 0.97 (3H, s, C_20_-CH_3_), 0.88 (1H, dd, *J* = 13.4, 5.2Hz, C_15_-CH_2_*_β_*), 0.79 (1H, m, C_1_-CH_2_*_β_*). The data of ^13^C-NMR was showed in Table S2. HREIMS *m/z* 359.2187 [M + Na]^+^ (calcd for C_20_H_32_O_4_Na, 359.2186).

**Table S2** The data of ^13^C-NMR of compounds **1-4**.

| **NO.** | **Comp. 1** | **Comp. 2** | **Comp. 3** | **Comp. 4** |
| --- | --- | --- | --- | --- |
| **C-1** | 40.68 | 40.15 | 40.15 | 40.40 |
| **C-2** | 18.95 | 18.74 | 18.74 | 18.56 |
| **C-3** | 37.81 | 37.58 | 37.58 | 31.74 |
| **C-4** | 44.88 | 42.77 | 42.77 | 49.41 |
| **C-5** | 56.77 | 55.88 | 55.88 | 50.98 |
| **C-6** | 22.31 | 21.93 | 21.93 | 21.99 |
| **C-7** | 41.02 | 41.85 | 41.85 | 41.19 |
| **C-8** | 43.25 | 43.97 | 43.97 | 44.33 |
| **C-9** | 55.17 | 55.36 | 55.36 | 55.51 |
| **C-10** | 39.38 | 39.04 | 39.04 | 39.15 |
| **C-11** | 18.26 | 18.13 | 18.13 | 18.46 |
| **C-12** | 30.88 | 25.81 | 25.81 | 31.16 |
| **C-13** | 41.31 | 44.48 | 44.48 | 38.15 |
| **C-14** | 37.55 | 36.80 | 36.80 | 36.63 |
| **C-15** | 44.33 | 52.74 | 52.74 | 44.96 |
| **C-16** | 45.27 | 80.48 | 80.48 | 42.98 |
| **C-17** | 179.69 | 65.27 | 65.27 | 66.28 |
| **C-18** | 28.12 | 28.53 | 28.53 | 69.50 |
| **C-19** | 180.27 | 178.57 | 178.72 | 178.39 |
| **C-20** | 14.84 | 15.30 | 15.30 | 15.07 |

**Table S3** Statistical Table of quality control Results

| **Sample** | **Clean paired reads** | **Clean bases(G)** | **Q30(%)** | **GC content(%)** | **Clean data ratio(%)** |
| --- | --- | --- | --- | --- | --- |
| M1 | 6893178 | 2.04 | 98.18 | 37.58 | 94.9 |
| M2 | 7045358 | 2.09 | 98.05 | 36.96 | 93.9 |
| M3 | 6333526 | 1.87 | 98.12 | 37.8 | 94.24 |
| C1 | 6804186 | 2.02 | 97.99 | 36.68 | 94.56 |
| C2 | 6241215 | 1.84 | 98.1 | 39.17 | 93.33 |
| C3 | 6133115 | 1.81 | 97.92 | 40.22 | 91.88 |

Sample: Sample name; M1-3: MRSA group, C1-3: compound 1 group; Clean paired reads: the total number of paired reads after quality control; Clean bases(G): total data; Q20 (%): the percentage of bases with more than 99% correct identification rate; Q30 (%): the percentage of bases with a correct identification rate above 99.9%; GC content (%): the percentage of the number of G and C bases in the total number of bases; Clean data ratio (%): The ratio of Clean bases to total bases.
